# Culturing hypoxia-primed mesenchymal stem cells in xeno- and serum-free conditions facilitates the synthesis of an extracellular matrix-based biologic with augmented therapeutic potential for the treatment of diabetic wounds

**DOI:** 10.1186/s13287-025-04827-z

**Published:** 2025-12-02

**Authors:** Kwok Keung Lit, Cheuk Kwan Owen Li, Zhamilya Zhirenova, Anna Blocki

**Affiliations:** 1 Institute for Tissue Engineering and Regenerative Medicine, The Chinese University of Hong Kong, Hong Kong SAR, China; 2 School of Biomedical Sciences, Faculty of Medicine, The Chinese University of Hong Kong, Hong Kong SAR, China; 3 Center for Neuromusculoskeletal Restorative Medicine (CNRM), Hong Kong Science Park, New Territories, Shatin, Hong Kong SAR China; 4 Department of Orthopaedics & Traumatology, Faculty of Medicine, The Chinese University of Hong Kong, Hong Kong SAR, China

**Keywords:** Xeno-free/serum-free, Hypoxic culture, Extracellular matrix, Revascularization, Diabetic wound healing

## Abstract

Diabetic wound healing is severely impaired by poor angiogenesis, neuropathy, chronic inflammation, decreased cell proliferation and dysregulated extracellular matrix (ECM) synthesis. Here, we developed a human mesenchymal stem cell (MSC)-derived ECM-based biologic with augmented pro-angiogenic potential, free of any xeno- and serum-free components, aimed at treating diabetic wounds. Hypoxia-primed MSCs, cultured in chemically defined medium, synthesized a secretome enriched in pro-angiogenic factors. This secretome was aggregated and co-precipitated by a heparan sulfate mimetic, dextran sulfate (DxS), into the pericellular space. MSCs assembled the deposited secretome components into an insoluble ECM, which upon decellularization was processed into MIcroParticles of SOlidified Secretome (MIPSOS), an ECM-DxS composite biologic. This formulation of MIPSOS demonstrated augmented pro-angiogenic bioactivity in vitro and in vivo and accelerated revascularization, re-epithelization and wound closure by almost doubling wound closure rate, and outperforming GraftJacket^®^, a clinically utilized decellularized human dermis-derived biologic. MIPSOS-treated wounds showed enhanced cellular infiltration and granulation tissue formation, along with a reduced presence of macrophages, indicating progression toward inflammation resolution. MIPSOS thus provides a novel treatment alternative that overcomes major limitations of soluble factor-based, tissue-derived ECM-based, as well as cell-based therapies, offering significant improvements in diabetic wound management.

**Supplementary Information:**

The online version contains supplementary material available at 10.1186/s13287-025-04827-z.

## Introduction

Impaired re-vascularization is one of the key pathophysiological features of chronic diabetic wound healing [1]. Due to the resulting poor tissue perfusion, ischemic tissues are continuously injured by low oxygen tension and nutrient deprivation, leading to prolonged inflammation, increased oxidative stress, dysregulated extracellular matrix (ECM) synthesis, decreased cell proliferation and migration, as well as impaired re-epithelization [2]. It is therefore essential to re-establish sufficient blood supply to the affected tissues.

Various therapeutic approaches have been developed to promote angiogenesis in ischemic wounds [3–5]. Unfortunately, they suffer from limited clinical efficacy and are associated with risks and side effects. Growth factor therapies aim to supply exogenous pro-angiogenic factor(s) to promote re-vascularization. Yet, these soluble factors have short half-lives and high diffusion rates, thus are required to be supplied in high doses, resulting in risks of carcinogenesis and off-site effects [6-8]. Moreover, the provision of a single or few factors lacks the necessary biocomplexity to effectively orchestrate and regulate angiogenesis [9]. Cellular therapies, such as mesenchymal stem cell (MSC) therapies, represent a more holistic approach, where MSCs promote angiogenesis and regeneration through a wide range of secreted factors [10, 11]. However, the inflamed and oxygen-deprived host environment hinders the survival and engraftment of MSCs, while the low rate of cell retention limits the clinical efficacy of MSC therapies [12]. Tissue-derived decellularized ECM-based biomaterials have been a more effective option, as the inherent complex topographical cues and biochemical signals of the indigenous scaffold promote and facilitate a spectrum of cellular activities [13]. Nonetheless, such ECM-based materials are acquired by decellularization of human or animal cadaveric tissues, while the bioactive composition cannot be altered to meet the desired bioactivity [14]. In addition, the use of cadaveric sources and incomplete removal of immunogenic components carries the inherent risk of disease transmission and undesirable immune responses [15, 16]. As an alternative, researchers have developed various biomaterials that mimic some features of the ECM, including topography, degradability, and these materials are often supplemented with exogenous bioactive factors [17, 18]. However, these biomaterials only resemble the tissue ECM to a limited extent and thus cannot match the therapeutic efficacy of ECM-based materials [19, 20].

Given these limitations, prior research has focused on utilizing ECM derived from in vitro culture, to supplement wound patches or hydrogels for application to skin wounds. Indeed, ECM derived from fibroblasts or MSCs improved wound healing by promoting revascularization, M2 macrophage infiltration, and granulation tissue formation. However, wound closure accelerated typically only minimally, if at all [21–23], suggesting that the potential of cell-derived ECM has not been harnessed fully in the past.

Our team has previously developed an approach to synthesize a biologic based on MSC-derived ECM in vitro, termed MIcroParticles of Solidified Secretome (MIPSOS) [24]. MSCs secrete a wide range of pro-angiogenic and pro-reparative factors [25] and are efficient ECM producers, while MSC-produced ECM at least partially retains the beneficial intrinsic properties of tissue ECM, such as biocomplexity. The biomaterial is synthesized by culturing human bone marrow MSCs (BM-MSCs) in the presence of ascorbate and dextran sulfate (DxS). DxS is a non-toxic sulfated polyglucose, and DxS of 500 kDa was illustrated to aggregate and co-precipitate ECM proteins into the pericellular space, thereby facilitating ECM assembly [21]. Additionally, this macromolecule is also a heparan sulfate mimetic, known to bind bioactive factors [26]. Indeed, we have shown that ECM co-assembly with DxS led to an accumulation of bioactive molecules within the biomaterial [23, 24]. The resulting DxS-ECM composite material can then be decellularized, extracted and lyophilized into fine insoluble fragments ranging from nm to µm sizes, termed MIPSOS. MIPSOS exhibited superior pro-angiogenic properties in vitro and in vivo and accelerated healthy skin wound healing in a pre-clinical mouse model, exceeding the therapeutic potential of naïve MSC-derived ECM, synthesized in the absence of DxS [24].

Nonetheless, the initial MIPSOS synthesis process utilizes foetal bovine serum (FBS). FBS and other serum-derived products are known for their high batch-to-batch variability in terms of quality and composition [27], potentially compromising the bioactivity of the synthesized ECM, thus impacting treatment efficacy. In addition, animal serum components have a high likelihood of being incorporated into the synthesized ECM [21], resulting in unwanted immunogenic responses upon implantation [28].

We thus sought to synthesize MIPSOS under controlled xeno- and/or serum-free conditions to create an MSC-derived ECM-based biologic composed of human-origin components, while simultaneously enhancing its pro-angiogenic capacity. Given that culture conditions, such as the choice of medium [29] and low oxygen tension [30, 31], are known to promote MSCs to secrete elevated levels of pro-angiogenic factors, MIPSOS were generated using various commercially available xeno- and/or serum-free media, to ensure enhanced reproducibility and safety, and optionally under hypoxia (5% O₂). These MIPSOS variants were vetted in vitro for their pro-angiogenic potential and their therapeutic potential was investigated in vivo in a murine diabetic skin wound healing model. We hypothesized that the optimal choice of medium and hypoxic culture conditions will synergistically amplify the pro-angiogenic activity of MSC secretome, leading to an augmented pro-angiogenic potential of MIPSOS, mediated by the DxS-driven enrichment of bioactive factors within MSC-derived ECM. Upon application of MIPSOS, this enhancement was expected to translate into accelerated and improved diabetic wound healing, underscoring its therapeutic potential.

## Materials and methods

### Cell culture

Human BM-MSCs (Cat. No.: SCC034, Millipore Massachusetts, United States) and human umbilical vein endothelial cells (HUVECs) (Cat. No.: PCS-100-013, ATCC, Virginia, United States) were purchased, through local distributors, from Millipore and ATCC, respectively. BM-MSCs from a total of 5 different donors were utilized in the different experiments in this study (Details: Suppl. Fig. S1). BM-MSCs were expanded and cultured in Dulbecco’s Modified Eagle Medium (DMEM, with 1 g/L glucose and GlutaMAX) (Life Technologies, California, United States), supplemented with 10% Fetal Bovine Serum (FBS) (ExCell Bio, Shanghai, China) and 1% Penicillin/Streptomycin (P/S) solution (Life Technologies, California, United States). HUVECs from pooled donors were expanded and cultured in EGM-2 BulletKit (Cat. No.: CC-3162, Lonza, Basel, Switzerland). Cells below passage 8 were used in all experiments.

### ECM-based material synthesis

BM-MSCs were seeded at 7000 cells/cm^2^ overnight to allow attachment. On the next day expansion medium was replaced with ECM induction media. All ECM induction media contains 10 µg/ml DxS (500 kDa, Cat. No.: D8906S, Sigma-Aldrich, St. Louis, United States) and 30 µg/ml ascorbic acid (Cat. No.: A8960, Sigma-Aldrich, St. Louis, United States) in various media listed in Table 1.

**Table 1:** Culture media utilized for MSC culture and ECM synthesis

Culture media	Abbreviation	Xenofree	Serum-free	Chemically Defined
Dulbecco‘s Modified Eagle Medium supplemented with 0.5% Fetal Bovine Serum	DMEM/FBS	✕	✕	✕
CnT-Prime MSC Proliferation Medium, Xeno-Free (Cat. No.: CnT-PR-MSC-XF, CELLnTEC Advanced Cell Systems AG, Bern, Switzerland)	CnT	✓	Low human- serum	NA
MSC NutriStem^®^ XF Medium (Cat. No.: 05-200-1 A, Sartorius, Göttingen, Germany) supplemented with PLTGold Human Platelet Lysate, research grade (Cat. No.: PLTGOLD100R, Sartorius, Göttingen, Germany)	NutriStem	✓	Medium is serum-free, but supplement contains human platelet lysate	✓
Mesenchymal Stem Cell Growth Medium XF (Cat. No.: C-28019, PromoCell, Heidelberg, Germany)	Promocell	✓	✓	NA
StemXVivo Xeno-Free Human MSC Expansion Media (Cat. No.: CCM021, R&D Systems, Minnesota, United States)	StemXVivo	✓	✓	NA
R: Stem (Cat. No.: EM1-500, Rohto, Osaka, Japan)	RStem	✓	✓	✓

Cells were cultured for 6 days, with no change of medium, in separate incubators maintained at normoxia (21% O_2_), and hypoxia (5% O_2_), at 37°C, 5% CO_2_.

After 6 days of culture, cultures were either fixated or decellularised. Decellularisation was carried out by incubating the culture at room temperature for 15 min with 0.15% (w/V) sodium deoxycholate (Cat. No.: 30970, Sigma-Aldrich, St. Louis, United States), supplemented with 0.175% (V/V) protease inhibitor cocktail (Cat. No.: P8340, Sigma-Aldrich, St. Louis, United States) in deionized water. Residual DNA was removed by subsequent incubation at 37^◦^C for 15 min with 0.04 mg/ml DNAse I (Cat. No.: LS002007, Worthington Biochemical, Lakewood, United States) in 1x Dulbecco′s Phosphate Buffered Saline with calcium chloride and magnesium chloride (Cat. No.: D1283, Sigma-Aldrich, St. Louis, United States). The resulting material was washed with deionized water, air dried and stored at 4◦C for short term or -20◦C for long term. Materials were left undisturbed on well-plates for all in vitro experiments. For in vivo experiments, the residual materials were collected mechanically with a cell scraper into deionized water, resulting in the formation of ECM fragments. ECM-fragments containing solution was then lyophilized into fine powder.

### Metabolic activity and viability assays

BM-MSC metabolic activity and viability were assessed by CCK8 assay and Live/Dead cell staining, respectively, according to the manufacturer’s protocols. In brief, the spent media were replaced by fresh medium (DMEM, 10% FBS, 1% P/S) supplemented with Cell Counting Kit 8 (Cat. No.: K1018, ApexBio, Houston, United States). The absorbance of the medium was measured at 450 nm after 2 h of incubation at 37°C, 5% CO_2_. Separate cultures were stained with LIVE/DEAD™ Viability/Cytotoxicity Kit for Mammalian Cells (Cat. No.: L3224, Invitrogen, Life Technologies, United States), and imaged using an epifluorescence microscope (IX83, Olympus, Tokyo, Japan).

For HUVEC metabolic activity, cells were seeded onto the decellularized matrices at density of 7000 cells/cm^2^ and cultured for 3 days at 37°C, 5% CO_2_ in EGM-2. Activity was then assayed as described above.

### Immunocytochemistry

All cultures were either fixed with ice-cold methanol for 20 min at -20^◦^C, or with 4% Paraformaldehyde (PFA) for 30 min at room temperature. Non-specific binding sites were blocked with 3% bovine serum albumin (BSA) in 1x Phosphate Buffer Saline (PBS), with or without 0.025% Triton X-100, for 1 h at 4^◦^C. The cultures were subsequently incubated with primary antibodies in 1% BSA in 1x PBS overnight at 4^◦^C, followed by 2-hour incubation at room temperature with corresponding secondary antibodies in 1% BSA in 1x PBS. Images were taken using an epifluorescence microscope (IX83, Olympus, Tokyo, Japan; ECLIPSE Ti2-A Imaging System, Nikon, Tokyo, Japan), and analyzed using ImageJ software. Images were analyzed by converting the images to 8-bit binary format, and manual binary thresholding was applied to segregate the positive signal for measurements. Percentage area coverage can then be quantified using the “Measure” function of ImageJ, after selecting the “Area Fraction” parameter in the “Set Measurement” menu.

All antibodies and dyes used are listed in Table 2.

**Table 2:** Antibodies used for immunocytochemistry

	Host	Dilution	Catalog Number	Manufacturer
**Primary Antibodies**				
Col I	Mouse	1:700	C2456	Sigma
Fibronectin	Rabbit	1:500	ab2413	abcam
HIF-1-α	Rabbit	1:200	20960-1-AP	Proteintech
VEGF-A	Mouse	1:200	66828-1-lg	Proteintech
Cytokeratin 1 (K1)	Mouse	1:50	ab9286	abcam
CD31	Goat	1:50	AF3628	R&D Systems
CD206	Rabbit	1:200	24,595	R&D Systems
iNOS	Rabbit	1:20	PA1-036	Thermofisher
F4/80	Rat	1:200	71,299 S	Cell Signalling Technology
**Secondary Antibodies**				
Anti-Rabbit 488	Donkey	1:500 (ICC) 1:200 (IHC)	ab150061	abcam
Anti-Mouse 488	Goat	1:500 (ICC)	ab150113	abcam
Anti-Rabbit 555	Donkey	1:500 (ICC)	ab150066	abcam
Anti-Mouse 555	Goat	1:500 (ICC) 1:200 (IHC)	ab150118	abcam
Anti-Rat 555	Donkey	1:200 (IHC)	ab150134	abcam
Anti-Rabbit 647	Donkey	1:200 (IHC)	ab150075	Abcam
Anti-Goat 647	Donkey	1:200 (IHC)	A-21,447	Invitrogen
**Probes**				
Phalloidin-iFluor 555		1:1000	ab176756	abcam
Phalloidin-iFluor 647		1:1000	ab176759	abcam
DAPI		1:1000	62,247	ThermoFisher

IF – immunofluorescence; Suppliers: Sigma-Aldrich, St. Louis, United States; Abcam, Cambridge, United Kingdom; Proteintech, Illinois, United States; Thermo Scientific™, Massachusetts, United States)

### Endothelial cell spheroid sprouting assay

Endothelial cell spheroids were generated by seeding HUVECs into spheroid-forming plates (AggreWell™400, Cat. No.: 34415, Vancouver, Canada) at 700 cells/micro-well using fully supplemented EGM-2 medium and incubation at 37^◦^C, 5% CO_2_, overnight. Spheroids were collected in EGM-2 and then mixed 1:1 with EGM-2 containing Collagen I solution (Cat. No.: 5225, Advanced BioMatrix, California, United States) at final concentration of 1 mg/ml. The spheroid-collagen mixture was gently casted onto the matrices and the collagen hydrogel was allowed to polymerize for 2 h at 37^◦^C, after which fresh EGM-2 was overlayed. Spheroids were allowed to sprout for 2 days, before they were fixed with 4% PFA and stained for F-actin using the above mentioned Phalloidin-iFluor 555 probe. The spheroids were subsequently imaged using an epifluorescence microscope (ECLIPSE Ti2-A Imaging System, Nikon, Tokyo, Japan) and their sprout length determined using ImageJ software.

### Tail skin wound model

All animal experiment procedures were approved by the Animal Experimentation Ethics Committee, The Chinese University of Hong Kong. 6–8 weeks old male diabetic db/db mice (*n* = 8 per condition) were used. Sample size was decided based on the number of animals used in the previous study [24], factoring in a 10% infection risk and thus exclusion from the study. Mice were housed in acryl cages at 4 animals per cage in a light-controlled room with a 12:12 h light/dark cycle with *ad libitum* access to water and rodent chow. A total of 40 mice were used. Animals were randomly assigned to various treatments and no blinding was performed. The dorsal side of the diabetic db/db mouse tail was chosen as a wound location, as it allows for sufficient surface area for critical-size wounds, while the tail lacks the subcutaneous panniculus carnosus muscle layer and thus heals without contraction. ^32^ For creation of the wound, mice were anaesthetized using continuous isoflurane. Following the negative paw reflex, confirming deep sedation, two 1 cm x 0.3 cm, spaced 0.5 cm apart, full-thickness wounds were created on the tails of mice to reduce required animal numbers [32]. The different MIPSOS materials were delivered in a fibrinogen solution and polymerized in situ. Fibrin hydrogels were prepared by mixing thrombin (Cat. No.: 605190, Sigma-Aldrich, St. Louis, United States) at final concentration of 5 U/ml in 0.9% NaCl and fibrinogen (Cat. No.: 341576, Sigma-Aldrich, St. Louis, United States) at a final concentration of 10 mg/ml in 0.9% NaCl. Fibril hydrogels were utilized as delivery vehicles and applied to animals as the control group. For this, fibrin hydrogels were prepared briefly before topical application into the wounds to enable the polymerization of a stable fibrin matrix upon application into wounds. For the experimental groups, different cell-derived ECM-based microparticles (21% O2 DMEM/FBS MIPSOS, 5% O2 DMEM/FBS MIPSOS, 5% O2 R: Stem MIPSOS), prepared as described above, and GraftJacket^®^ (Wright Medical Group, Inc., United States) was ground into microfragments. All materials were suspended in fibrinogen solutions. Upon addition of thrombin, polymerizing solutions were applied to the wounds. The two wounds on each mouse were treated with different conditions to minimize inter-individual response variation. The wounds were covered with transparent film dressing. Animal postoperative wellbeing was ensured in accordance with protocols as approved by the institution Animal Experimentation Ethics Committee.

The size of the wounds was monitored by imaging the wounds at fixed distances across different time points. Wound edges were visually identified as a boundary of the pinkish healed flesh, and the surface area of the remaining wound was measured. Scab areas were treated as wound areas. Rate of healing of each wound on each mouse was calculated using simple regression analysis model by GraphPad Prism v10.0 (GraphPad Software, California, United States).

Animals were euthanized at the endpoint using carbon dioxide asphyxiation. The animals were placed in an air-tight chamber and100% carbon dioxide was injected at a fill rate of about 30% to 70% of the chamber volume per minute until the animals were unconscious (around 4 min). Death was confirmed by examining the animal for cessation of vital signs, such as lack of pulse and breathing, response to firm toe pinch, and rigor mortis.

### Histology and immunohistochemistry

Whole tails of the mice were harvested at the end of the experiment, after animals were euthanized by carbon dioxide. Tails were fixed with 4% PFA for 1 day, before being transferred into 14% Ethylenediaminetetraacetic acid (EDTA) solutions. Tails in EDTA were incubated at 37°C for 7 days, with daily changes of EDTA solution, to achieve decalcification of the tail bone. Resulting tissues were embedded in paraffin and 10 μm sections were prepared. Sections were stained with hematoxylin & eosin (H&E), Masson’s trichrome and immunostained for markers listed above. For immunofluorescence staining, sections were dewaxed and antigen retrieval was performed in citrate buffer, followed by 3 × 10 min washes with 0.025% Triton X-100 in 1x Tris Buffer saline (TBS) on gentle agitation. Sections were subsequently blocked with blocking solution (0.3 M glycine and 3% Bovine serum albumin (BSA) in 1x TBS) for 1 h in room temperature. Sections were then incubated with primary antibodies in 1% BSA in 1x TBS overnight in 4°C, and secondary antibodies in 1% BSA in 1x TBS for 2 h at room temperature, with 3 × 10 min washes with 0.025% Triton X-100 in 1x TBS on gentle agitation after each of the antibody incubation steps. Whole tail section images were taken using an epifluorescence microscope (ECLIPSE Ti2-A Imaging System, Nikon, Tokyo, Japan; ECLIPSE Ti2-E Imaging System, Nikon, Tokyo, Japan) using a 10x or 20x objective lens with large image acquisition, where images taken were automatically stitched by the Nikon NIS-Element image acquisition software using a 10% image overlap definition.

All histology images were analyzed using ImageJ software. Granulation tissue in each section was visually identified as the area with high cell density and dense background indicating high extracellular matrix composition. One representative region of interest (ROI) was selected from each of the sections (*n* = 4 per condition) for analysis. In brief images were first deconvoluted using the ImageJ “color deconvolulation2” plugin tool, where the in-built vector for each combination dye staining in the tool, as validated by the developer (https://blog.bham.ac.uk/intellimic/g-landini-software/colour-deconvolution-2/), decomposes the images into single color channel corresponding to single dye staining. Manual binary thresholding was applied to segregate nuclei from the deconvoluted haematoxylin images, which were then counted using the “analyze particle” function of ImageJ. Mason Trichrome images were analyzed using the histogram function of ImageJ to extract the mean intensity of the blue signal. Immunostained images were analyzed as described above.

### Data presentation and statistical analysis

All statistical analysis was performed using one- or two-way ANOVA with GraphPad Prism v10.0 (GraphPad Software, California, United States) with p values less than 0.05 considered as statistically significant. No blinding was done in any part of the experiments. All data were assumed equal variance, except for the spheroid sprouting experiment where Shapiro–Wilk test was used to test for data normality. Kruskal-Wallis test was performed for statistical analysis for the non-normally distributed data. All in vitro experiments were performed at least as three biologically independent repeats, while 8 animals per condition were used for wound area quantification and 3 animals per condition for histological examination for in vivo experiments. Data are presented as mean +/- standard deviation. All schematics were generated with BioRender (www.biorender.com).

## Results

### MIPSOS can be synthesized under xeno- and/or serum-free conditions

**Fig. 1:**
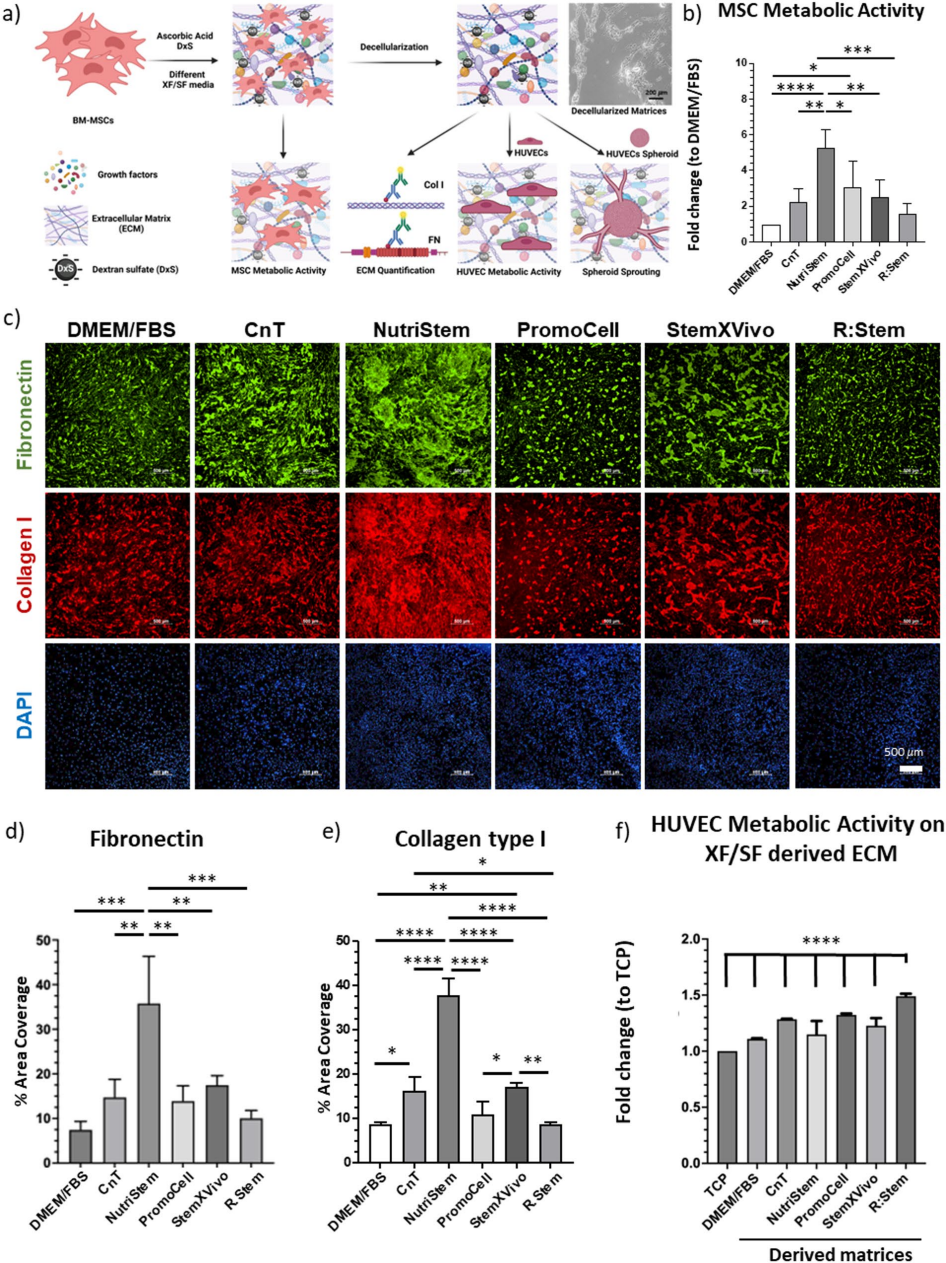
Synthesis and evaluation of MSC-derived ECM-based substrates deposited in various cell culture media. (a) Schematic workflow of ECM synthesis and evaluation (b) Quantification of MSC metabolic activity cultured in various XF/SF media. Data are displayed as fold-changes compared to DMEM/FBS. (Measurements for individual biological repeats (*n* = 4) can be found in Suppl. Fig. S2). (c) Representative immunocytochemistry images for fibronectin and collagen I deposited by MSCs, when cultured in various cell culture media. Scale bar = 500 μm. (d and e) Quantification of area coverage of stained fibronectin and collagen I, respectively, as depicted in c). (f) Investigation of HUVEC metabolic activity on decellularized MSC-derived ECM-based substrates, synthesized in various media. Data are displayed as fold-changes compared to TCP. Only significant differences as compared to matrices synthesized in R: Stem are displayed. (Measurements for individual biological repeats (*n* = 6) and full statistical information can be found in Suppl. Fig. S2b) * *p* < 0.05, ** *p* < 0.01, *** *p* < 0.001, **** *p* < 0.0001. Graphs represent the average reading from each of a minimum of *n* = 3 biological repeats. Error bars represent mean ± SD

In order to produce MIPSOS under xeno- and/or serum-free conditions, BM-MSCs were cultured in several commercially available xeno-free (XF) and/or serum-free (SF) media (Table 1) in the presence of DxS and ascorbate at previously established concentrations for 6 days (Fig. 1a). All media supported MSC maintenance over the time period of ECM synthesis. Indeed, MSC metabolic activity was enhanced in the chosen media, suggesting 2 to 5-fold higher cell yields after 6 days (Fig. 1b, Suppl. Fig. S2a).

In order to determine whether the amount of deposited ECM was affected by the various media, MSC cultures were stained for two major ECM structural components collagen I and fibronectin and the stained area was quantified. Culturing MSCs in NutriStem medium most significantly promoted the deposition of both ECM components, whereas cells cultured in other media exhibited comparable amounts of deposited collagen I and fibronectin as in DMEM/FBS cultures (Fig. 1c-e). In fact, quantities of collagen I and fibronectin exhibited similar trends as was noted for MSC metabolic activity in the various media (Fig. 1b-e). Noteworthy, ECM components in all cultures exhibited a granular pattern, as previously reported for ECM deposited in the presence of DxS. ^24,33^ Upon successful decellularization (Suppl. Fig. S3a), the resulting matrices were evaluated for their pro-angiogenic properties by examining their ability to support endothelial cell proliferation. For this, HUVECs were seeded on decellularized matrices and cell metabolic activity was determined by CCK8 assay after 3 days. All matrices synthesized in the presence of XF/SF media promoted HUVEC metabolic activity, suggesting enhanced cell proliferation as compared to cultures on tissue culture polystyrene (TCP). Hence, all media choices appeared to be compatible with DxS-driven ECM deposition and to exhibit enhanced endothelial cell supporting properties. Remarkably, HUVECs cultured on matrices synthesized in the R: Stem medium exhibited the statistically significantly highest metabolic activity (Fig. 1f, Suppl. Fig. S2b). These results were unexpected, as R: Stem medium did not promote ECM deposition as effectively as the other XF/SF media (Fig. 1c-e), suggesting that amounts of deposited ECM did not necessarily correlate with the extent of endothelial-supporting bioactivity of the biologic and that ECM deposited in the presence of R: Stem may be enriched in signaling factors promoting endothelial proliferation. Taking into consideration that R: Stem-derived matrices promoted endothelial cell metabolic activity most efficiently, indicative of enhanced proliferation, R:Stem was chosen for further experiments.

### Hypoxia and R: stem medium synergistically augment pro-angiogenic properties of synthesized MIPSOS

**Fig. 2:**
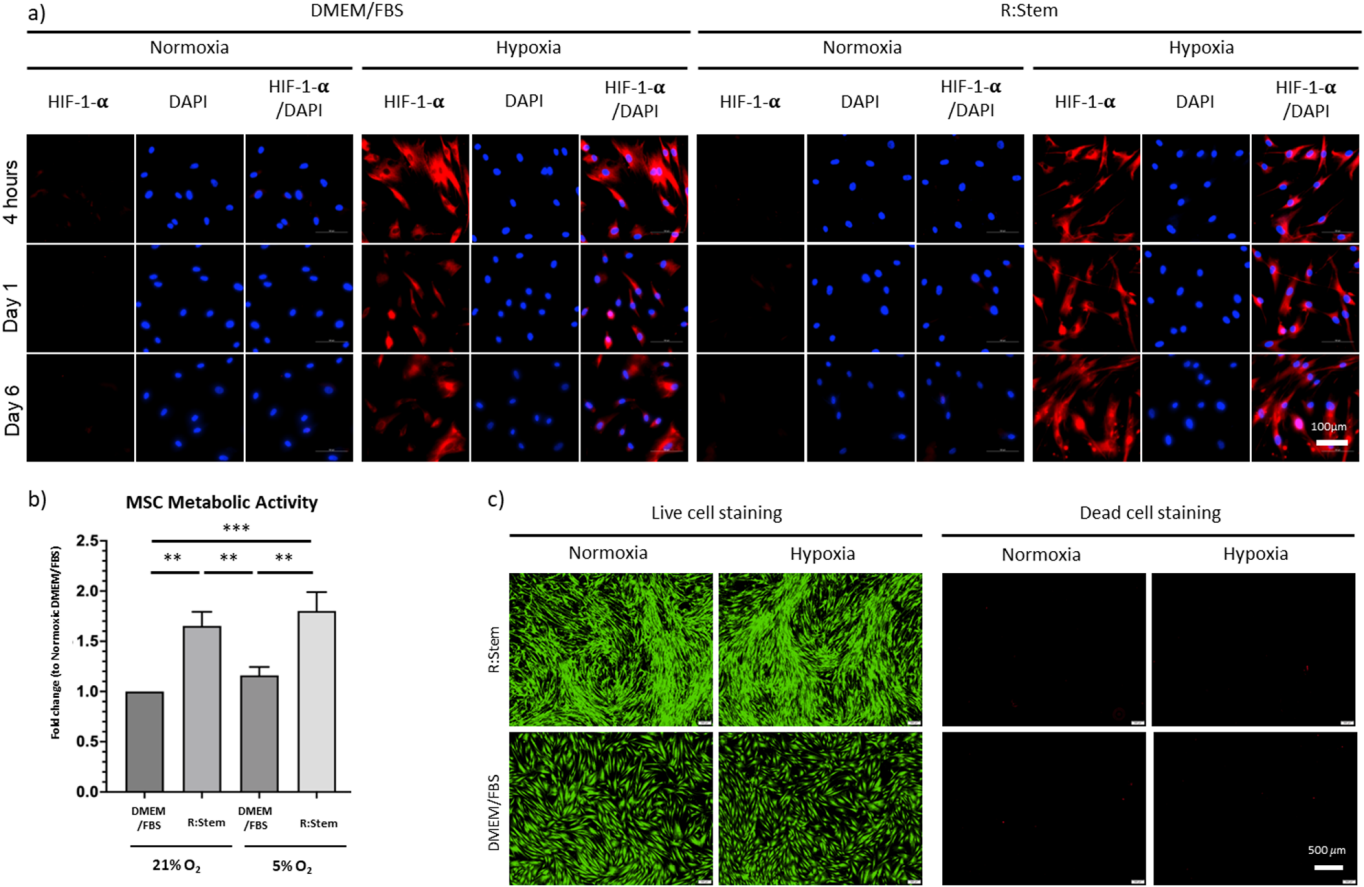
MSCs cultured under low oxygen tension were viable and exhibited comparable metabolic activity to normoxic cultures. (a) Immunocytochemistry staining for HIF-1-α in MSCs cultured in DMEM/FBS or R: Stem under hypoxic or normoxic conditions for up to 6 days. Nuclei were stained with DAPI. Scale bar = 100 μm (b) Assessment of MSC metabolic activity by CCK-8 assay after 6 days of culture. Data are displayed as fold-changes compared to DMEM/FBS. (Measurements for individual biological repeats (*n* = 3) can be found in Suppl. Fig. S4a) (c) Live/Dead cell staining of MSCs after 6 days of culture. Scale bar = 500 μm. *, *p* < 0.05; **, *p* < 0.01; ***, *p* < 0.001; ****, *p* < 0.0001. Graphs represent average reading from each of a minimum of *n* = 3 biological repeats. Error bars represent mean ± SD

Culturing MSCs under hypoxic conditions was reported to protect transcription factor hypoxia-inducible factor 1-alpha (HIF-1-α) from degradation, facilitating its translocation to the nucleus, thereby driving the transcription of angiogenesis-related genes [34]. This would in turn result in an increased secretion of pro-angiogenic factors [31, 35], which we anticipated to be incorporated into their ECM during assembly and thus hypothesized that the resulting MIPSOS assembled under hypoxic conditions would enhance the pro-angiogenic potential of the biologic. Successful stabilization of HIF-1-α was confirmed in MSCs cultured under low oxygen tension, independent of the culture medium utilized, throughout the whole period of ECM deposition of 6 days (Fig. 2a). In contrast, no HIF-1-α was observed in MSCs cultured under normoxic conditions. MSC cultures exhibited significantly increased metabolic activity when grown in R: Stem as compared to DMEM/FBS, while hypoxic cell culture had no further effect (Fig. 2b, Suppl. Fig. S4b). Similarly, Live/Dead cell staining confirmed visibly increased cell density in R: Stem cultures, while no cell death was observed in any of the cultures (Fig. 2c), suggesting that choice of medium and hypoxia had no adverse effects on cell viability.

**Fig. 3:**
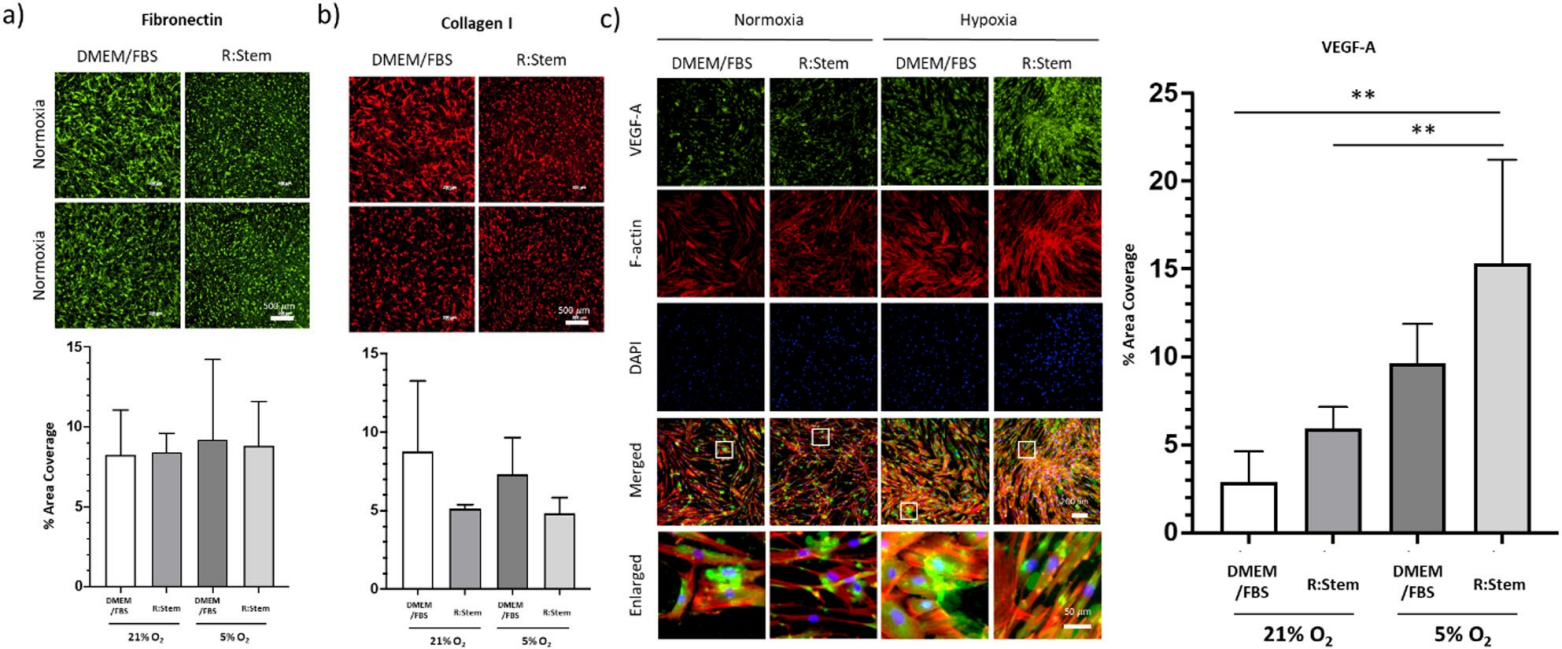
Hypoxia and R: Stem synergistically increase VEGF-A amounts in MSC cultures. Immunocytochemistry staining and quantification of stained area coverage for (a) fibronectin, (b) collagen I, Scale bar = 500 μm, and (c) immunocytochemistry staining and quantification of stained area coverage for VEGF-A, (Representative area outlined by white lines are enlarged and presented below each image) Scale bar = 200 μm, and 50 μm (Enlarged images), in MSCs cultured in DMEM/FBS or R: Stem under hypoxic or normoxic conditions for 6 days. *, *p* < 0.05; **, *p* < 0.01; ***, *p* < 0.001; ****, *p* < 0.0001. Graphs represent average reading from each of a minimum of *n* = 3 biological repeats. Error bars represent mean ± SD

Upon successful establishment of hypoxic cultures, the effect of hypoxia on ECM deposition was evaluated. Immunocytochemical evaluation of cell cultures revealed no significant differences for deposited structural ECM components fibronectin (Fig. 3a) and collagen I (Fig. 3b). Staining for vascular endothelial growth factor A (VEGF-A), a downstream target of HIF-1-α activation [36], revealed elevated VEGF-A levels in MSCs cultured in R: Stem as compared to DMEM/FBS, which was further enhanced by hypoxic conditions for both media types (Fig. 3c). Enlarged imaged areas further revealed VEGF-A positively stained areas intracellularly (overlapping with staining of cytoskeleton) and extracellularly exhibiting a granular pattern in all cultures, suggesting the successful incorporation of this ECM-bound factor [37] into the DxS-enhanced matrix.(Fig. 3c). Quantification of positively stained areas demonstrated higher levels of VEGF-A in R: Stem over DMEM/FBS medium, which was further enhanced by hypoxic culture conditions in the respective media. Hence, highest VEGF-A levels were observed in R: Stem medium under hypoxic conditions.

**Fig. 4:**
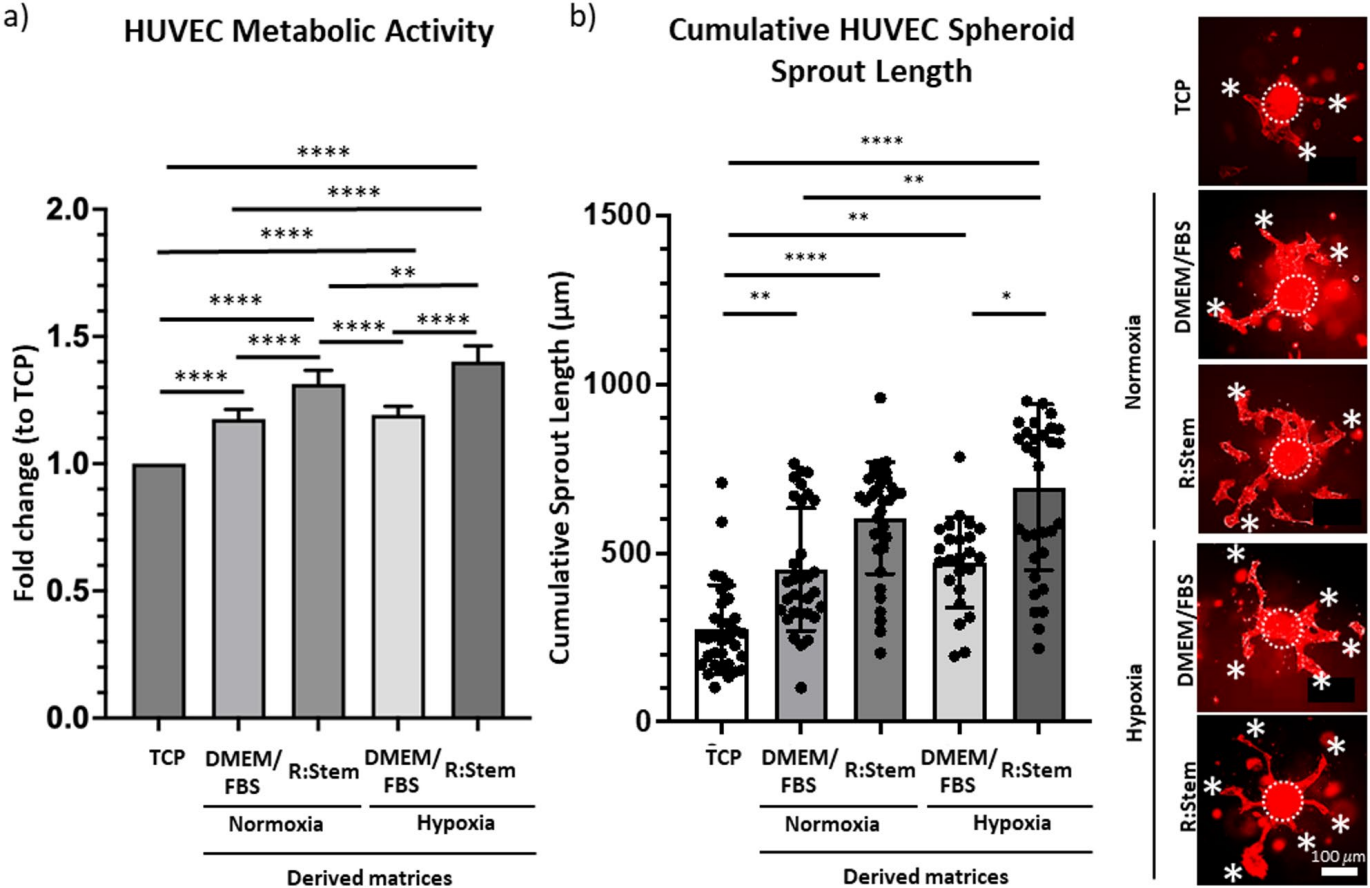
Hypoxia and R: Stem medium synergistically augment pro-angiogenic potential of MSC-derived matrices. (a) HUVECs were seeded on decellularized matrices assembled in DMEM/FBS or R: Stem under normoxic or hypoxic conditions and their metabolic activity was evaluated by CCK-8 assay after 3 days. Data are displayed as fold-changes compared to TCP. (Measurements for individual biological repeats (*n* = 6) can be found in Suppl. Fig. S4b) (b) HUVEC spheroids were embedded in collagen I hydrogels and overlaid over matrices assembled in DMEM/FBS or R: Stem under normoxic or hypoxic conditions and allowed to sprout for 2 days. Samples were stained with phalloidin, imaged and measured cumulative sprouting length was plotted on a graph. *, *p* < 0.05; **, *p* < 0.01; ***, *p* < 0.001; ****, *p* < 0.0001. Graphs represent the average reading from each of a minimum of *n* = 3 biological repeats. Error bars represent mean ± SD

When decellularized matrices (Suppl. Fig. S3b) were seeded with HUVECs, all materials significantly promoted endothelial metabolic activity, as compared to TCP, suggesting enhanced proliferation. As expected, both DMEM/FBS and R: Stem-derived ECM-based materials synthesized under hypoxia were able to significantly promote endothelial metabolic activity as compared to their respective counterparts synthesized under normoxic conditions (Fig. 4a, Suppl. Fig. S4b). Interestingly, materials synthesized using R: Stem under normoxia promoted endothelial metabolic activity more efficiently than matrices assembled in DMEM/FBS and under hypoxia, suggesting that choice of medium is of high relevance, when synthesizing ECM-based materials with specific bioactivities. When endothelial spheroids were seeded directly onto decellularized matrices in collagen I hydrogels, a significantly longer cumulative sprout length per spheroid was observed on all matrices as compared with TCP, with the most significant increase in cumulative sprout length on matrices assembled in R: Stem and under hypoxia. Again, the medium of choice for ECM assembly had the most prominent effect on the pro-angiogenic potential of matrices, as matrices synthesized in R: Stem outperformed matrices assembled in DMEM/FBS (Fig. 4b). Unexpectedly, hypoxia seemed not to have such a profound effect on the pro-angiogenic potential of ECM-based materials, as only slight increments were noted in in vitro assays.

### MIPSOS synthesized under defined conditions and under hypoxia most efficiently accelerated diabetic wound healing in a mouse tail wound model

**Fig. 5:**
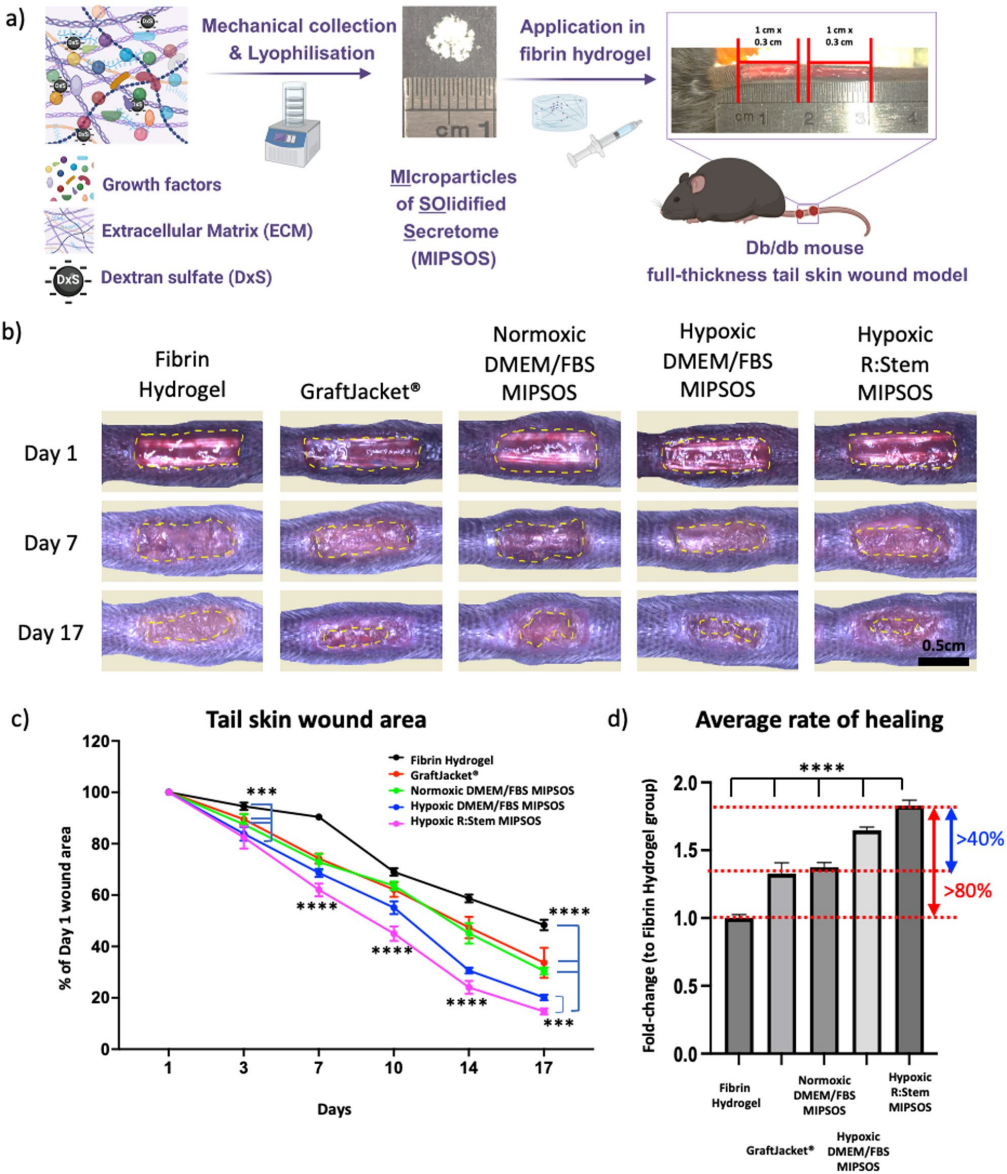
MIPSOS synthesized under hypoxia in R: Stem medium most efficiently accelerated diabetic wound healing. (a) Schematic workflow of MIPSOS preparation and application into full-thickness skin tail wounds of db/db mice (b) Representative images of wounds over a time course of 17 days. Yellow dashed lines indicate the wound outline. Scale bar = 0.5 cm (Images of wounds at all time points can be found in Suppl. Fig. S5) (c) Measured wound areas plotted over time. Only statistical significances compared to Hypoxic R: Stem MIPSOS are displayed. All statistical information can be found in Suppl. Fig. S5. (d) Average healing rate quantified from wound areas over time. Data are displayed as fold-changes compared to Fibrin hydrogel. All groups displayed significant differences of *p* < 0.0001 from all other group, except from GraftJacket^®^ and normoxic DMEM MIPSOS treatment group which were not significantly different from each other. * *p* < 0.05, ** *p* < 0.01, *** *p* < 0.001, **** *p* < 0.0001. Graphs represent the average reading from *n* = 8 animals. Error bars represent mean ± SD

Based on our preliminary *in vitr*o findings and to reduce the number of required animals for experimentation, we refined our in vivo study design. Hence, MIPSOS synthesized using DMEM/FBS, under normoxic or hypoxic conditions and MIPSOS synthesized in R: Stem under hypoxic conditions were investigated for their therapeutic efficacy in full-thickness skin wounds in diabetic mice (Fig. 5a). For this, the various MSC-derived matrices were mechanically scraped into deionized water and freeze-dried to obtain MIPSOS as typical insoluble microfragments. The dorsal side of the diabetic db/db mouse tail was chosen as a wound location, as it allows for sufficient surface area for critical-size wounds, while the tail lacks the subcutaneous panniculus carnosus muscle layer and thus heals without contraction [32]. Two wounds were created on the tail of each mouse (Fig. 5a). The different MIPSOS materials were delivered in a fibrinogen solution and polymerized by thrombin in situ. A clinically approved human dermal tissue-derived ECM (GraftJacket^®^), known to exhibit good clinical outcome in the treatment of chronic wounds [38], was chosen as a clinical gold standard for comparison. GraftJacket^®^ was broken into microfragments and added at the same concentration into wounds.

As expected, untreated (vehicle only) wounds exhibited a slow healing capability, resulting in a 50% wound closure after 17 days. Wounds treated with GraftJacket^®^ and the original MIPSOS had comparable healing and demonstrated an acceleration of wound closure from day 3 onwards, resulting in 65% wound closure by day 17 (Fig. 5b and c, Suppl. Fig. S5). These effects were even more pronounced for MIPSOS synthesized under hypoxic conditions, with MIPSOS assembled in R: Stem significantly outperforming MIPSOS assembled in DMEM/FBS from day 7 onwards (Fig. 5b and c, Suppl. Fig. S5). As a result, by day 17, wounds treated with MIPSOS synthesized under hypoxic conditions in DMEM/FBS or R: Stem closed by 80% and 85%, respectively (Fig. 5b and c, Suppl. Fig. S5). Simple linear regression analysis of wound sizes over time revealed that hypoxic R: Stem MIPSOS accelerated wound closure by 80% and 40% compared to vehicle and GraftJacket^®^ treatment groups, respectively (Fig. 5d).

**Fig. 6:**
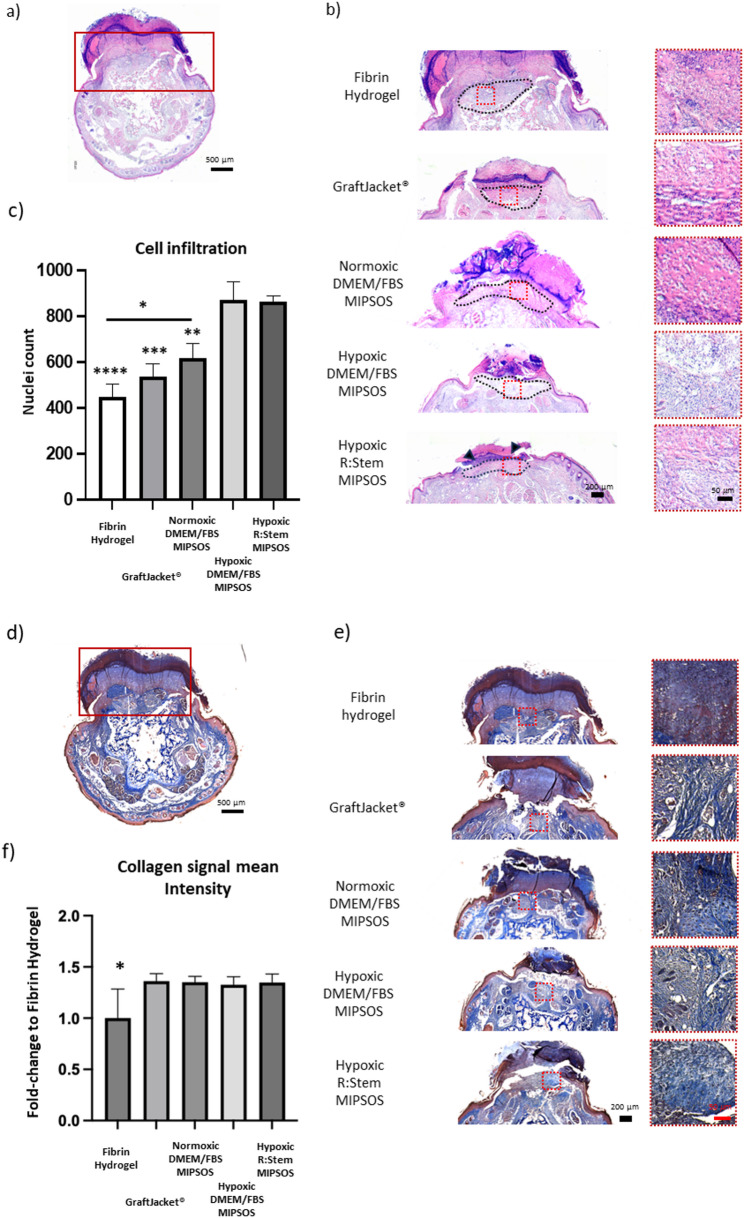
Diabetic wounds treated with MIPSOS synthesized under hypoxic conditions exhibit the most advanced healing. (a) Representative H&E staining of whole tail cross-section. Scale bar = 500 μm (b) H&E staining of tail sections harboring wounds. Scale bar = 200 μm. Granulation tissue is outline by black dashed lines and selected areas (red dashed squares) are enlarged on the right side. Scale bar = 50 μm (c) Semi-quantitative analysis of hematoxylin stained nuclei in H&E stained granulation tissues. Significances without bars indicate differences to each of the hypoxia derived MIPSOS. (d) Representative Masson’s Trichrome staining of whole tail cross-section. Scale bar = 500 μm (e) Masson’s Trichrome staining of tail sections harboring wounds. Scale bar = 200 μm. Selected areas of granulation tissue (red squares) are enlarged on the right side. Scale bar = 50 μm (f) Semi-quantitative analysis of blue-stained collagen fibers in Masson’s Trichrome stained granulation tissues. Significances without bars indicate differences to all other conditions displayed. Data are displayed as fold-changes compared to fibrin hydrogel only. *, *p* < 0.05; **, *p* < 0.01; ***, *p* < 0.001; ****, *p* < 0.0001. Graphs represent the average reading from each of *n* = 3 tissue samples. Error bars represent mean ± SD

Whole cross-sections of day 17 decalcified mouse tails harboring wounds were histologically evaluated. H&E staining of tail cross-sections (Fig. 6a), allowed clear visualization of granulation tissue (Fig. 6b, area outlined by black dashed lines). Cellular infiltration into granulation tissue was semi-quantitatively assessed in close-ups of granulation tissue areas (Fig. 6b, red squares). MIPSOS synthesized under hypoxia, regardless of the medium used, promoted the most pronounced cellular infiltration by day 17, even outperforming the clinically approved GraftJacket^®^ (Fig. 6c).

Masson’s Trichrome staining of whole tail cross-sections (Fig. 6d) allowed a clear visualization of collagen rich granulation tissues (Fig. 6e). Semi-quantitative analysis of collagen content demonstrated an increased collagen density in all treated wounds, as compared to the vehicle control, there was, however, no difference observed between the various treatments (Fig. 6f).

**Fig. 7:**
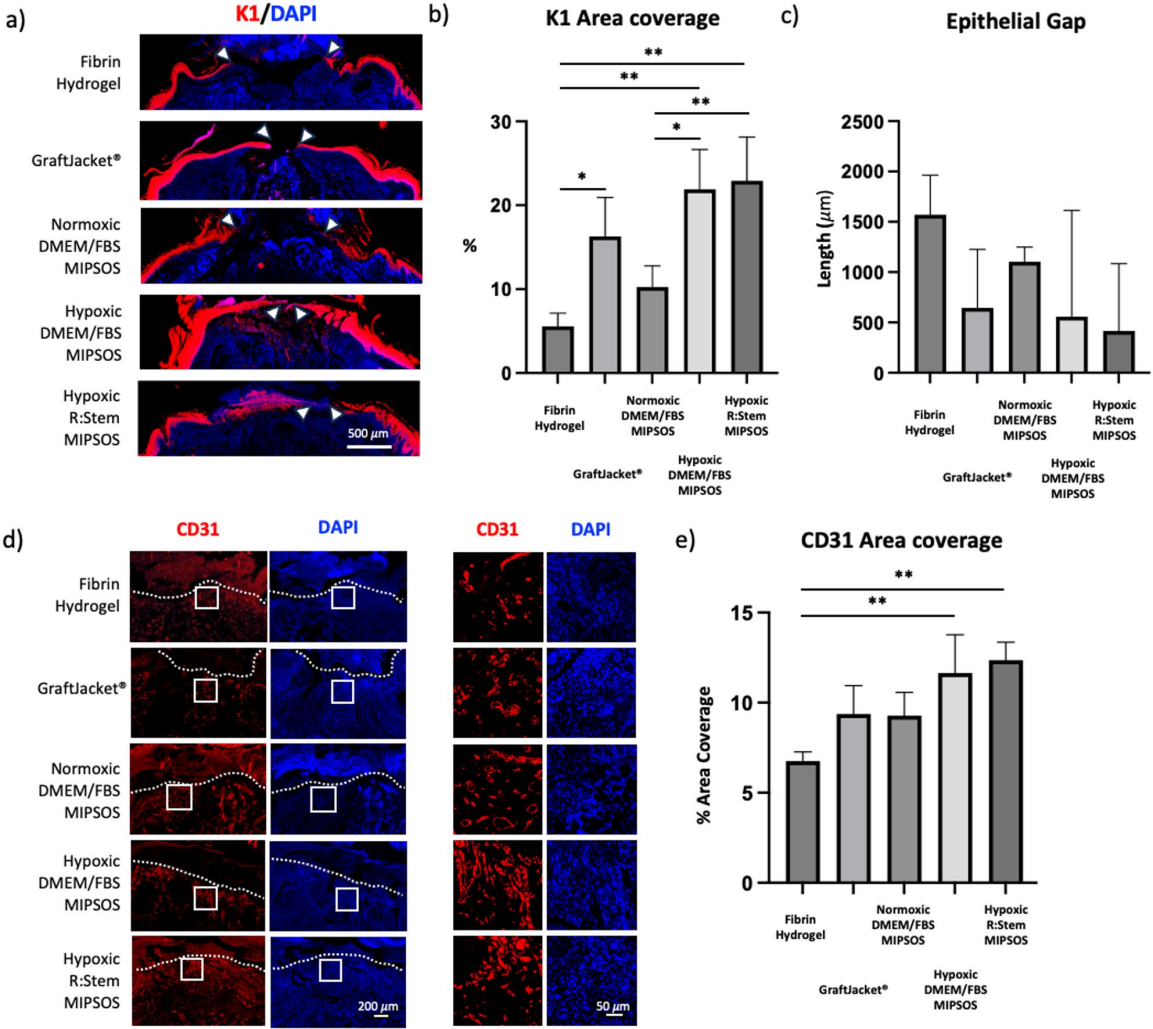
MIPSOS promoted re-epithelialization and re-vascularization of diabetic wounds. (a) Immunofluorescence staining of day 17 wounds for cytokeratin 1 (K1) and DAPI. Scale bar = 500 μm (b) Semi-quantitative assessment of K1 area coverage and (c) the remaining epithelial gap on day 17. (d) Representative images of CD31 and DAPI staining of day 17 wound. Interface between wound and scab area is indicated by white dashed line. Selected areas, as outlined by the white box, are enlarged and displayed on the right. Scale bar = 200 μm (overview pictures, left) and 50 μm (enlarged view, right) (e) Quantification of area stained positively for CD31 per field of view (FOV), respectively. *, *p* < 0.05; **, *p* < 0.01; ***, *p* < 0.001; ****, *p* < 0.0001. Error bars represent mean ± SD

Immunohistochemical analysis of cytokeratin I (K1), a marker for mature epidermis [39], demonstrated close to full re-epithelialization of wounds treated with MIPSOS synthesized under hypoxic conditions in DMEM/FBS and R: Stem. Comparable and partial re-epithelialization was observed for wounds treated with MIPSOS produced under original conditions (normoxia & DMEM/FBS) and GraftJacket^®^, while only limited epithelial growth was observed in wounds treated with the vehicle only (fibrin hydrogel) (Fig. 7a). Semi-quantitative analysis of K1 positive tissue area and epithelial gap width demonstrated corresponding trends. Although high variability led to no significant differences in gap closure in between groups, quantification of K1 positively stained areas indicated thicker and more pronounced re-epithelialization in wounds treated with MIPSOS synthesized under hypoxic conditions (Fig. 7b and c). Of note, K1 immunostaining confirmed that re-epithilialization took place underneath the scab, suggesting that wound closure was even further progressed as assessed macroscopically (Fig. 5).

Semi-quantitative analysis of immunohistochemical staining of granulation tissue for CD31 revealed that all materials significantly promoted higher vessel density as compared to the vehicle group, with wounds treated with MIPSOS synthesized under hypoxia, regardless of the choice of medium, exhibiting the highest endothelial cell density (Fig. 7d and e).

**Fig. 8:**
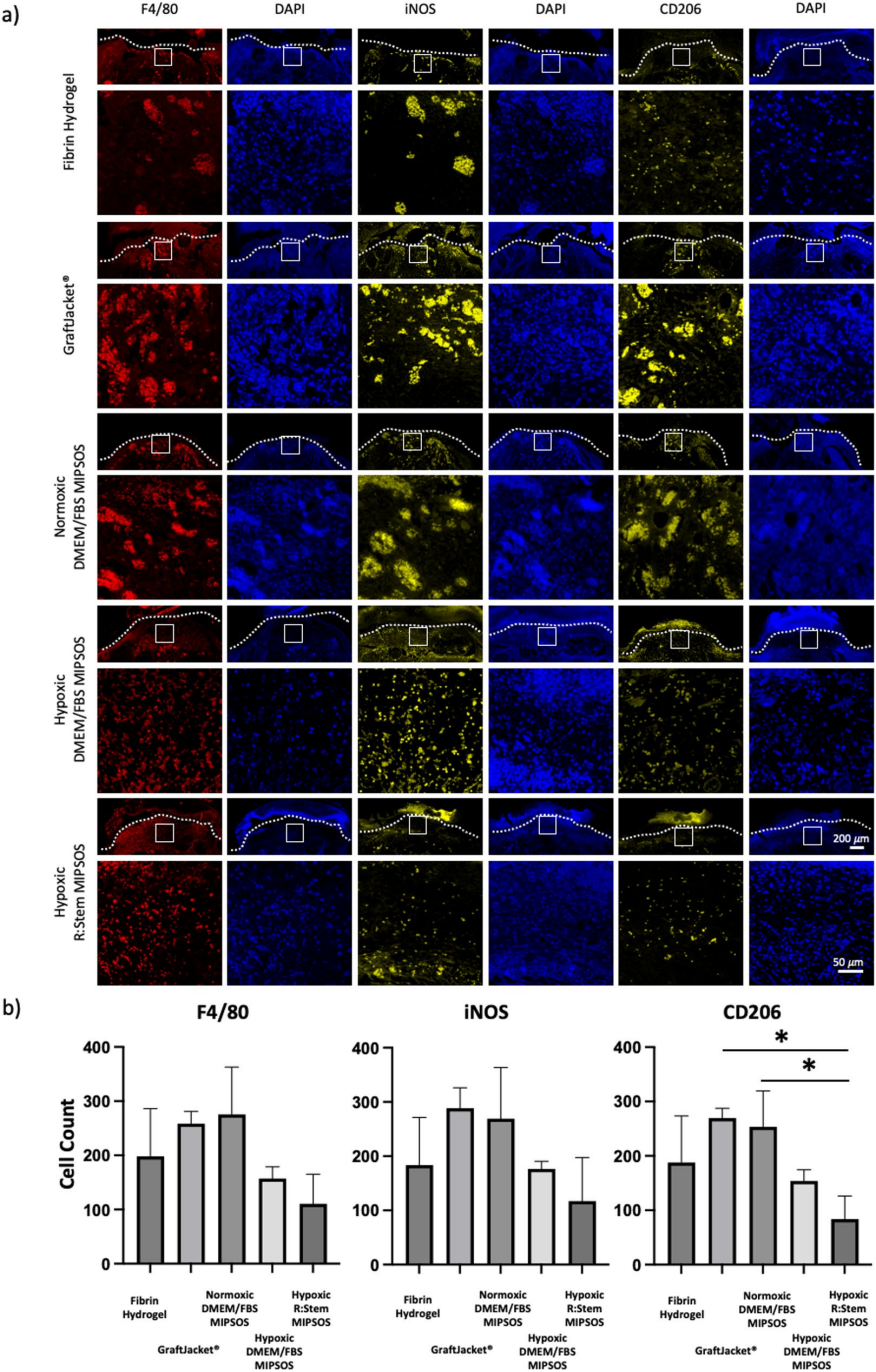
Wounds exhibit variable levels of macrophages. (a) Representative images of F4/80, iNOS and CD206 stained wounds on day 17. Interfaces between wound and scab areas are indicated by white dashed lines. Selected areas, as outlined by the white box, are enlarged and displayed below. Scale bar = 200 μm (overview pictures, top) and 50 μm (enlarged view, below) (b) Quantification of cell stained for F4/80, iNOS and CD206 per field of view (FOV), respectively. *, *p* < 0.05; **, *p* < 0.01; ***, *p* < 0.001; ****, *p* < 0.0001. Graphs represent average reading from each of a minimum of *n* = 3 tissue samples. Error bars represent mean ± SD

As an increased cellular infiltration was noted in all ECM treated groups (Fig. 6a-c), this could be attributed to an increased presence of macrophages, an indicator of ongoing inflammation [40]. We thus stained tissue sections for mouse macrophage marker F4/80, as well as for iNOS^+^ and CD206^+^, M1 and M2 markers, respectively. Surprisingly, although semi-quantitative analysis of F4/80^+^ cells showed a slight increase in cell numbers in the granulation tissues of wounds treated with GraftJacket^®^ and MIPSOS synthesized with DMEM/FBS under normoxia as compared to that by vehicle only, the numbers were reduced when treated with materials synthesized under hypoxia (Fig. 8a and b). A similar trend was observed for iNOS^+^ and CD206^+^ stained cells, respectively. These data suggested that inflammation further resolved in wounds treated with MIPSOS synthesized under hypoxia and that the observed increased presence of cells as observed in Fig. 6a-c, can be likely attributed to fibroblasts and endothelial cells (Fig. 7e).

## Discussion

The combination of hypoxia, a chemically defined medium and the heparan sulfate mimetic, DxS, enabled the synthesis of MIPSOS with augmented pro-angiogenic potential and pro-healing bioactivity, significantly exceeding the therapeutic efficacy of a clinically approved tissue-derived ECM biologic (GraftJacket^®^), a gold-standard for the treatment of diabetic wounds. Indeed, in a diabetic wound healing model the improved MIPSOS preparation almost doubled the wound closure rate. This is a significant improvement to the results published in previous studies that utilized fibroblast or MSC-derived ECM and demonstrated only minimally enhanced wound closure rates of 10–20%, if any [21–23].

Recent reports highlighted an enhanced therapeutic potential of an MSC-derived ECM sheet, which was synthesized under chemically induced hypoxia using Cobalt (II) chloride (CoCl2). This procedure also enriched pro-angiogenic bioactive factors within the ECM, which subsequently promoted wound healing in healthy, albeit immunocompromised mice [31]. In contrast to our approach, no macromolecules were added to enhance ECM deposition, and ECM was derived only after weeks-long culture. Furthermore, the therapeutic effect of this MSC-derived ECM-based material remains to be determined on chronic and non-contracting wounds in immune-competent animals.

All XS/SF media tested supported MSC metabolism and ECM deposition similarly or even better than DMEM/FBS, while all media compositions were compatible with DxS supplementation, which is crucial for the synthesis of an ECM-based biologic with augmented pro-angiogenic properties [24] and highlights the general compatibility of DxS-driven ECM deposition with various culture conditions. Collagen I and fibronectin were chosen to assess the amounts of deposited ECM, as they, as structural components, comprise a large fraction of deposited ECM. Congruent with previous reports [41], hypoxia did not affect ECM deposition, while the choice of media had a significant effect. Nonetheless, deposited amounts of ECM did not allow to predict the strength of the ECM’s bioactivity, suggesting that the presence or amounts of other bioactive components may not necessarily correlate with the amounts of these structural ECM components. Here, ECM-based materials in R: Stem exhibited the highest pro-angiogenic potential, as indicated by their ability to promote endothelial proliferation.

Indeed, MSCs cultured in R: Stem synthesized more VEGF-A, a pan pro-angiogenic factor [42, 43], which was further enhanced under hypoxia. VEGF-A accumulated in granular ECM structures, which were previously identified as aggregates of cell-derived ECM and DxS [23, 24]. Indeed, DxS is known to act as a heparan sulfate mimetic and thus bind VEGF [44], besides others [45, 46], while it was shown to promote the accumulation of a wide plethora of pro-angiogenic factors in cell-derived ECM [24]. It can thus be assumed that the appropriate choice of culture conditions, which promote the synthesis of pro-angiogenic factors, act synergistically with DxS to facilitate their accumulation within cell-derived ECM.

Nonetheless in in vitro functional assays, the choice of culture medium enhanced the bioactivity of MSC-derived ECM most pronouncedly, while its synthesis under hypoxia held only minimal benefits for its pro-angiogenic potential. This contrasted with observations found in vivo, where ECM synthesis under hypoxia most efficiently promoted cell infiltration, enhanced re-vascularization and re-epithelialization, irrespective of media choice. Interestingly, the increased cell infiltration in both hypoxic MIPSOS groups correlated with the lowest number of macrophages, independent of macrophage polarization phenotype, suggesting a more advanced resolution of the inflammatory state. Since cell migration and infiltration is severely impaired in diabetic wound healing [47], the increased density of infiltrated cells in conjunction with a decreased inflammation is suggestive of a more advanced healing progression. Nonetheless, further investigation of the role of MIPSOS on macrophage phenotype and inflammation at intermediate time point remains necessary.

Advanced healing progression was further evidenced by wounds treated with MIPSOS exhibiting a denser *de novo* deposited ECM, a process known to be normally impaired during diabetic wound healing [48]. It cannot be excluded, however, that the observed pro-angiogenic and pro-reparative potential of MIPSOS is partially caused by components from the XF/SF medium being incorporated into the biologic. Nonetheless, MIPSOS synthesized under hypoxic conditions, independent if in DMEM/FBS or R: Stem, performed similarly in vivo. This suggests that MIPSOS contains an array of pro-regenerative factors, which are responsible for its profound therapeutic potential, independent of the choice of medium it was synthesized in. As the exact composition of R: Stem is unknown to us, future studies will focus on expression profiles of MSCs in DMEM/FBS and R: Stem to assess how the chosen media affected the MSC secretome. The therapeutic potential of MIPSOS synthesized in alternative chemically defined media can give further insight into the contribution of selected media to the therapeutic effect of MIPSOS.

The improved manufacturing protocol for MIPSOS enabled the synthesis of a MSC-derived ECM-based biologic of human origin, reducing the risk of adverse immunogenic responses and disease transmission [49], thereby paving the way for its GMP-compatible production and clinical translation. It is noteworthy, that although the ECM-based biological was of human origin it was investigated for its therapeutic efficacy in an immune-competent mouse model. As ECM is highly conserved across species, immunological responses towards pure ECM-materials are limited [50, 51]. Nonetheless, incomplete decellularization, as is often the case with tissue-derived ECM based materials [16], can lead to immune reactions and even rejections. As cell-derived ECM is deposited as a thin 2-dimensional layer, it can be thoroughly and gently decellularized [52], highlighting good immune-compatibility of cell-derived ECM based materials, enabling its testing in immune-competent animal models.

We have also previously reported that MIPSOS, synthesized with the original formula using normoxia and DMEM/FBS, promoted revascularization and healing in skin wounds of healthy and immunocompetent mice. Here we show that the traditional MIPSOS had a comparable therapeutic potential to an FDA-approved human dermal ECM-derived material (GraftJacket^®^), while the improved MIPSOS formula augmented diabetic wound healing in a murine model, accelerating wound closure by 40% as compared to GraftJacket^®^. This can be potentially attributed to the accumulation of pro-regenerative healing factors within MIPSOS. GraftJacket^®^ is derived from human decellularized skin [51], a homeostatic tissue, not undergoing remodeling and thus likely containing lesser amounts signaling factors, which promote wound healing. In contrast, MIPSOS is based on ECM, the insoluble secretome of MSCs, which are known to secrete a wide plethora of factors that promote tissue healing and regeneration [10, 11]. The choice of culture conditions (culture medium and hypoxia) in combination with the DxS-driven enrichment of bioactive factors within MSC-derived ECM led to an augmented pro-regenerative potential of MIPSOS, resulting in its superior therapeutic potential.

Although GraftJacket^®^ has previously been reported to promote satisfactory clinical outcomes in chronic wound treatment [38], it faces limitations such as limited availability, risk of immunogenicity, batch-to-batch variability and the inability to tailor its bioactivity for desired properties [16]. These can be addressed by synthesizing biologics, such as MIPSOS, in a laboratory setting under highly controlled conditions allowing to carefully tailor their bioactivity. As such utilization of MIPSOS for chronic wound treatment overcomes many of the challenges faced by tissue-derived biologics, such as GraftJacket^®^.

In contrast to utilizing the established technology to deliver ECM-rich cell sheets [53], removal of immunogenic cells enables the delivery of a simpler, off-the-shelf biologic, which is safer and broadly applicable in clinical settings. MIPSOS can retain its bioactivity for several weeks to months when stored frozen and desiccated, and is, therefore, a suitable off-the-shelf material that could become available to a large population of patients. It contains an enriched, complex portfolio of pro-reparative factors while being of human origin and cell-free. Its utilization can thus address major limitations of soluble factor-based, tissue-derived ECM-based, as well as cell-based therapies.

## Conclusion

The successful production of MIPSOS, with augmented pro-angiogenic properties, under controlled and xeno-free conditions has thus the potential to pave the way for its clinical translation for the treatment of non-healing chronic wounds. Future studies will focus on unravelling the underlying mechanism of action, defining appropriate quality controls and the upscaling of MIPSOS synthesis. Moreover, the core technology of synthesizing cell-derived ECM-based biologics in the presence of macromolecules [54–56] opens avenues to establish treatment approaches for other diseases and tissue injuries.

## Supplementary Information

Below is the link to the electronic supplementary material.

Supplementary Material 1

Supplementary Material 2

## Data Availability

All data can be provided upon reasonable request to the corresponding author.

## Abbreviations

BSA: Bovine Serum Albumin
CoCl2: Cobalt (II) chloride
DMEM: Dulbecco’s Modified Eagle Medium
DxS: Dextran sulfate
ECM: Extracellular matrix
FBS: Foetal bovine serum
GMP: Good manufacturing practices
HIF-1-α: Hypoxia-inducible factor 1-alpha
HUVEC: Human umbilical vein endothelial cells
K1: Cytokeratin 1
MSC: Mesenchymal stem cell
MIPSOS: MIcroParticles of Solidified Secretome
SF: Serum-free
TCP: Tissue culture polystyrene
VEGF-A: Vascular endothelial growth factor A
XF: Xeno-free
