# Supplementary material for: Culturing hypoxia-primed mesenchymal stem cells in xeno- and serum-free conditions facilitates the synthesis of an extracellular matrix-based biologic with augmented therapeutic potential for the treatment of diabetic wounds
Source: Stem Cell Res Ther. 2025 Dec 2;17:15. doi: 10.1186/s13287-025-04827-z (PMC12781806; doi:10.1186/s13287-025-04827-z)
Supplement: Supplementary file 2 — Supplementary Material 2 [file 13287_2025_4827_MOESM2_ESM.docx]

**Supplementary Information**

**Culturing Hypoxia-Primed Mesenchymal Stem Cells in Xeno- and Serum-Free Conditions Facilitates the Synthesis of an Extracellular Matrix-Based Biologic with Augmented Therapeutic Potential for the Treatment of Diabetic Wounds.**

**Authors**

Kwok Keung Lit ^1,2,3^; Cheuk Kwan Owen Li ^1,2^, Zhamilya Zhirenova ^1,2,3^ and Anna Blocki ^1,2,3,4 *^

* Correspondence: [anna.blocki@cuhk.edu.hk](mailto:anna.blocki@cuhk.edu.hk)

**Affiliations**

1 Institute for Tissue Engineering and Regenerative Medicine, The Chinese University of Hong Kong, Hong Kong SAR, China

2 School of Biomedical Sciences, Faculty of Medicine, The Chinese University of Hong Kong, Hong Kong SAR, China

3 Center for Neuromusculoskeletal Restorative Medicine (CNRM), Hong Kong Science Park, Shatin, New Territories, Hong Kong SAR, China

4 Department of Orthopaedics & Traumatology, Faculty of Medicine, The Chinese University of Hong Kong, Hong Kong SAR, China

| Result | Experiment performed | Internal Cell Donor ID and date of thawing | | | | | |
| --- | --- | --- | --- | --- | --- | --- | --- |
|  |  | Replicate 1 | Replicate 2 | Replicate 3 | Replicate 4 | Replicate 5 | Replicate 6 |
| Fig. 1b + Supp S1a | Different XF-MSC - CCK8 | WES (P6) 13-09-2021 | WES (P6) 13-09-2021 (2) | WES (P6) 20-09-2021 | WES (P6) 20-09-2021 (2) |  |  |
| Fig. 1c | Different XF-ECM - ICC | Zen (P7) 15-02-2023 | Zen (P6) 22-03-2023 | Zen (P7) 27-03-2023 |  |  |  |
| Fig. 1f + Supp S1b | Different XF-ECM HUVEC - CCK8 | YVES (P6) 15-02-2022 | YVES (P6) 15-02-2022 (2) | YVES (P6) 15-03-2022 | YVES (P6) 15-03-2022 (2) | YVES (P6) 15-03-2022 (3) | YVES (P6) 13-06-2022 |
| Fig. 2a | Hypoxia HIF1alpha - ICC | Iris (P7) 27-12-2023 | Iris (P7) 07-12-2023 | Iris (P6) 30-01-2024 |  |  |  |
| Fig. 2b + Supp S3a | Hypoxia MSC - CCK8 | Lou (P6) 15-03-2022 | Lou (P6) 15-03-2022 (2) | Lou (P6) 15-03-2022 (3) |  |  |  |
| Fig. 2c | Hypoxia MSC - Live/Dead | Lou (P6) 15-03-2022 | Lou (P6) 15-03-2022 (2) | Lou (P6) 15-03-2022 (3) |  |  |  |
| Fig. 3a + b | Hypoxia ECM - ICC (FN + Col I) | Zen (P6) 06-03-2023 | Zen (P6) 22-03-2023 | Zen (P6) 27-03-2023 |  |  |  |
| Fig. 3c | Hypoxia ECM - ICC (VEGFA) | Zen (P7) 27-12-2023 | Zen (P6) 30-01-2024 | Zen (P8) 09-04-2024 | Zen (P8) 10-12-2024 |  |  |
| Fig. 4a + Supp 3b | Hypoxia ECM - HUVEC CCK8 | Lou (P6) 15-03-2022 (2) | Lou (P6) 01-06-2022 | Lou (P6) 13-06-2022 | Lou (P6) 13-06-2022 (2) | Lou (P5) 29-08-2022 | Lou (P5) 29-08-2022 (2) |
| Fig. 4b | Hypoxia ECM - HUVEC Spheroid sprouting | Lou (P6) 01-06-2022 | Lou (P5) 29-08-2022 | Lou (P6) 30-11-2022 |  |  |  |
| Fig. 5-8 + Supp S4 | Animal experiments | Lou (P6) 14-11-2022 + Lou (P6) 30-11-2022 |  |  |  |  |  |
| Supp S2a | Different XF ECM - Decellularization | Zen (P7) 27-03-2023 | Zen (P7) 25-04-2024 | Zen (P8) 30-04-2024 |  |  |  |
| Supp S2b | Hypoxia ECM - Decellularization | Zen (P6) 13-06-2022 | Zen (P5) 28-02-2023 | Zen P5 28-03-2023 |  |  |  |

| Internal Cell donor ID | Lot number |
| --- | --- |
| Wes | 3221856 |
| Yves | 3221856 |
| Zen | 3735070 |
| Lou | 3602370 |
| Iris | 3602371 |

Supplementary Figure S1: Detailed information about donors used for each experimental repeat and date of thawing.


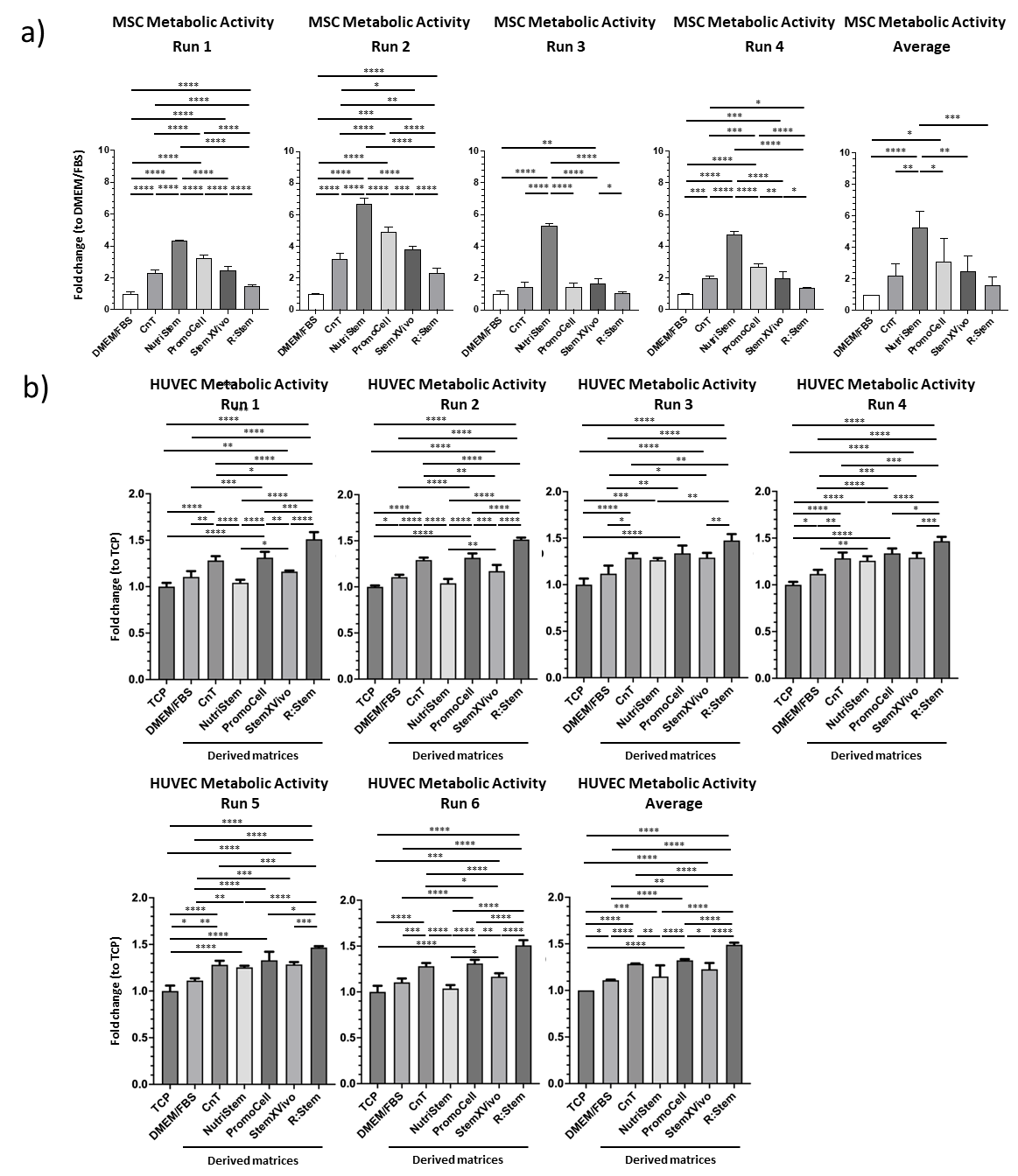


Supplementary Figure S2: Metabolic activity as determined by CCK8. Data are displayed for each biological replicate, as well as the average of all runs. (a) Quantification of MSC metabolic activity cultured in various XF/SF media after 6 days. Data are displayed as fold-changes compared to DMEM/FBS. (b) Investigation of HUVEC metabolic activity on decellularized MSC-derived ECM-based substrates, synthesized in various media after 3 days. Data are displayed as fold-changes compared to TCP. *, p<0.05; **, p < 0.01; ***, p < 0.001; ****, p < 0.0001. Error bars represent mean ± SD.


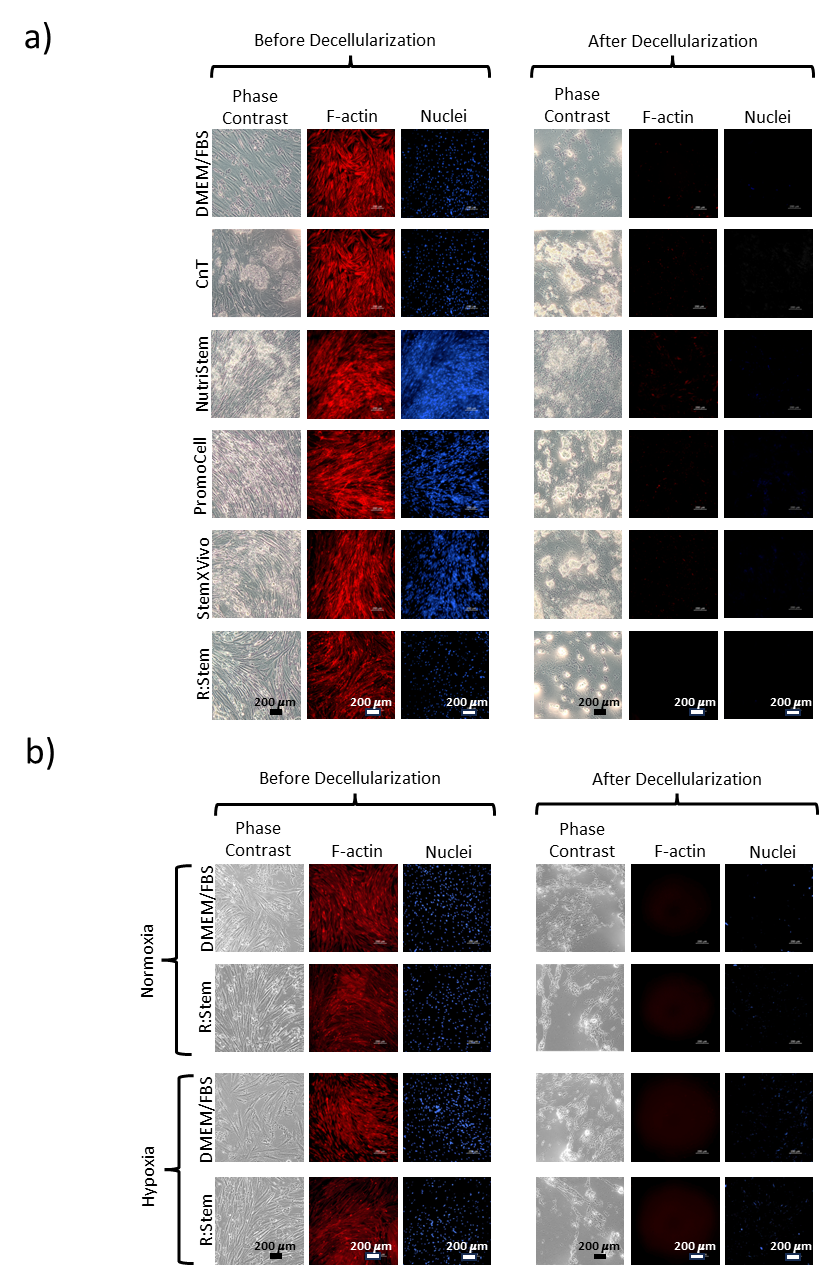


Supplementary Figure S3: Successful decellularization of MSC-derived matrices. MSC cultures were stained for F-Actin by phalloidin and their nuclei by DAPI and imaged by phase contrast before and after decellularization. a) MSCs cultured in various media (table 1). b) MSCs cultured in DMEM/FBS or R:Stem under normoxic or hypoxic conditions. Successful decellularization is confirmed by lack staining for cellular components F-actin and Nuclei, while phase contrast images clearly show network structures of remaining ECM. Scale bar = 200µm


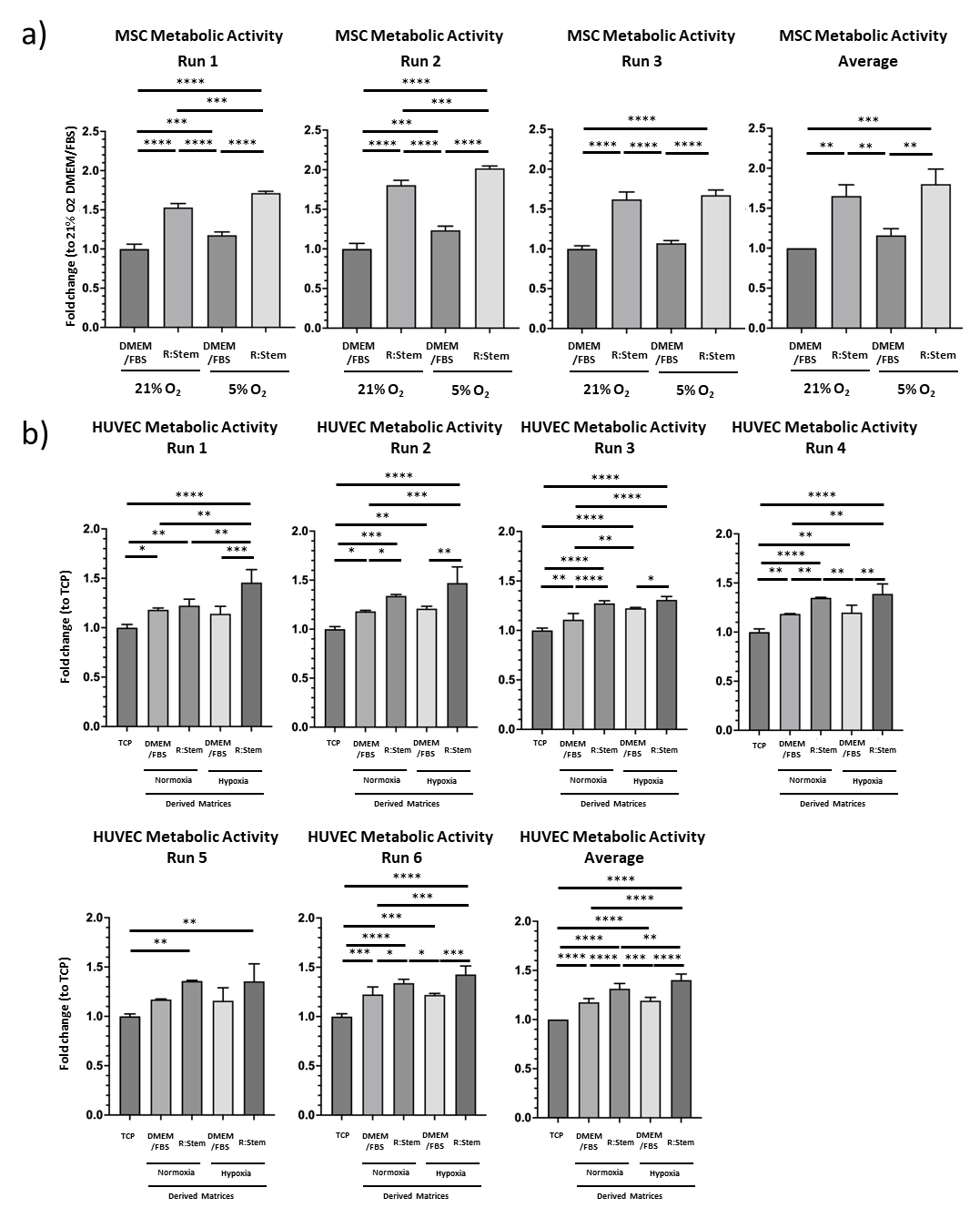


Supplementary Figure S4: Metabolic activity as determined by CCK8. Data are displayed for each biological replicate, as well as the average of all runs. (a) Assessment of MSC metabolic activity by CCK-8 assay after 6 days of culture. Data are displayed as fold-changes compared to DMEM/FBS. b) HUVECs were seeded on decellularized matrices assembled in DMEM/FBS or R:Stem under normoxic or hypoxic conditions and their metabolic activity was evaluated by CCK-8 assay after 3 days. Data are displayed as fold-changes compared to TCP. *, p<0.05; **, p < 0.01; ***, p < 0.001; ****, p < 0.0001. Error bars represent mean ± SD.


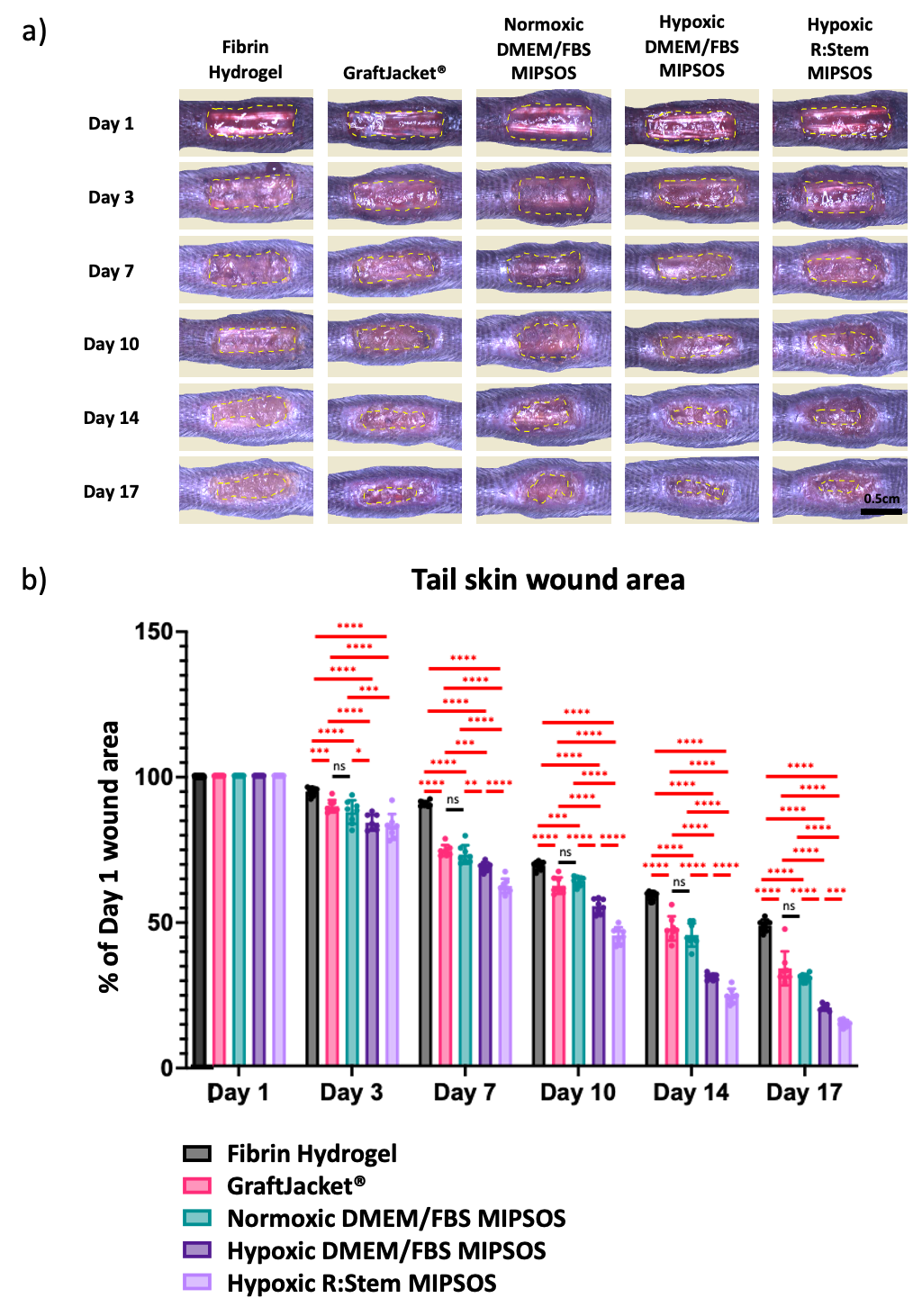


Supplementary Figure S5: MIPSOS synthesized under hypoxia in R:Stem medium most efficiently accelerated diabetic wound healing. (a) Representative images of wounds over a time course of 17 days. Yellow dashed lines indicate the wound outline. Scale bar = 0.5 cm (b) Measured wound areas as measured at various intermediate time points over time. *, p<0.05; **, p < 0.01; ***, p < 0.001; ****, p < 0.0001. Error bars represent mean ± SD.
